# Neuroinflammation and Neurodegeneration in Adult Rat Brain from Binge Ethanol Exposure: Abrogation by Docosahexaenoic Acid

**DOI:** 10.1371/journal.pone.0101223

**Published:** 2014-07-16

**Authors:** Nuzhath Tajuddin, Kwan-Hoon Moon, S. Alex Marshall, Kimberly Nixon, Edward J. Neafsey, Hee-Yong Kim, Michael A. Collins

**Affiliations:** 1 Department of Molecular Pharmacology & Therapeutics, Loyola University Chicago, Stritch School of Medicine, Maywood, Illinois, United States of America; 2 Department of Pharmaceutical Sciences, University of Kentucky, Lexington, Kentucky, United States of America; 3 Laboratory of Molecular Signaling, NIAAA, National Institutes of Health, Bethesda, Maryland, United States of America; Nathan Kline Institute for Psychiatric Research and New York School of Medicine, United States of America

## Abstract

Evidence that brain edema and aquaporin-4 (AQP4) water channels have roles in experimental binge ethanol-induced neurodegeneration has stimulated interest in swelling/edema-linked neuroinflammatory pathways leading to oxidative stress. We report here that neurotoxic binge ethanol exposure produces comparable significant effects *in vivo* and *in vitro* on adult rat brain levels of AQP4 as well as neuroinflammation-linked enzymes: key phospholipase A2 (PLA2) family members and poly (ADP-ribose) polymerase-1 (PARP-1). In adult male rats, repetitive ethanol intoxication (3 gavages/d for 4 d, ∼9 g/kg/d, achieving blood ethanol levels ∼375 mg/dl; “Majchrowicz” model) significantly increased AQP4, Ca**^+2^**-dependent PLA2 GIVA (cPLA2), phospho-cPLA2 GIVA (p-cPLA2), secretory PLA2 GIIA (sPLA2) and PARP-1 in regions incurring extensive neurodegeneration in this model—hippocampus, entorhinal cortex, and olfactory bulb—but not in two regions typically lacking neurodamage, frontal cortex and cerebellum. Also, ethanol reduced hippocampal Ca**^+2^**-independent PLA2 GVIA (iPLA2) levels and increased brain “oxidative stress footprints” (4-hydroxynonenal-adducted proteins). For *in vitro* studies, organotypic cultures of rat hippocampal-entorhinocortical slices of adult age (∼60 d) were ethanol-binged (100 mM or ∼450 mg/dl) for 4 d, which augments AQP4 and causes neurodegeneration (Collins et al. 2013). Reproducing the *in vivo* results, cPLA2, p-cPLA2, sPLA2 and PARP-1 were significantly elevated while iPLA2 was decreased. Furthermore, supplementation with docosahexaenoic acid (DHA; 22:6n-3), known to quell AQP4 and neurodegeneration in ethanol-treated slices, blocked PARP-1 and PLA2 changes while counteracting endogenous DHA reduction and increases in oxidative stress footprints (3-nitrotyrosinated proteins). Notably, the PARP-1 inhibitor PJ-34 suppressed binge ethanol-dependent neurodegeneration, indicating PARP upstream involvement. The results with corresponding models support involvement of AQP4- and PLA2-associated neuroinflammatory pro-oxidative pathways in the neurodamage, with potential regulation by PARP-1 as well. Furthermore, DHA emerges as an effective inhibitor of these binge ethanol-dependent neuroinflammatory pathways as well as associated neurodegeneration in adult-age brain.

## Introduction

Alcohol (ethanol) use disorders constitute a major worldwide cause of health impairment and productivity loss [1], [2]. Amidst a range of organ damage outcomes, chronic ethanol abuse—and particularly binge-type alcoholism [3], [4]—causes neuropathological sequelae leading to brain dysfunction and dementia [5]. A repetitive binge intoxication adult rat model developed by Majchrowicz nearly 40 years ago to study alcoholic withdrawal seizures [6] was later shown to have surprisingly regionalized neurodegeneration in the temporal (particularly entorhinal) cortex (EC) and the hippocampus (HC) (mainly dentate gyrus granule cells), as well as olfactory bulb neurons [7], [8]. Results indicating the involvement of oxidative stress but not excitotoxicity [9] in this *in vivo* model of acquired neurodegeneration point to neuroinflammatory (glial-neuronal) pro-oxidative mechanisms; however, the detailed pathways remain uncertain. The Majchrowicz adult rat intoxication model or a variant has been used by a number of laboratories to examine underlying mechanisms. Apoptosis as a chief neurodegenerative pathway was ruled out by the absence of TUNEL staining [10]. Evidence that diuretics such as furosemide and acetazolamide prevent ethanol-induced elevations in rat brain water content while reducing or blocking neurodegeneration indicated that brain edema was important [11], [12]. Cellular edema, a major and often intractable clinical problem that exacerbates neuropathology in brain ischemia and trauma, thus might elicit neuroinflammatory processes in binge ethanol abuse. A further contributor to the neurodamage in this binge model may well be stress-elevated circulating glucocorticoid (corticosterone), as recently reported [13]. (However, it is worth noting a paradox in that glucocorticoids have well-known edema-resolving effects, particularly under cerebral acidotic conditions [14]).

Neuroinflammation—brain pro-oxidative processes dependent on glia acting on neurons—has assumed a more general focus in alcohol neuropathology studies. While there is considerable interest in the role of microglial activation, it is unclear in the above binged rat model whether microglia contribute directly to neurodegeneration [15], [16]. Astroglia could have key roles, since they are enriched in membrane aquaporin-4 (AQP4) water channels that mediate glial edema during brain insults [17]. Furthermore, such brain astroglial swelling due to binge ethanol could promote phospholipid-instigated neuroinflammation—e.g., increased phospholipase A2 (PLA2) activities, which release excessive quantities of arachidonic acid (ARA, 20:4n-6) from membrane phospholipids [18], [19]. ARA can be a major source of reactive oxygen species (ROS) via autoxidation and its metabolism to eicosanoid products. Specific families of PLA2 known to mobilize ARA during brain insults and neuroinflammatory stresses were the focus of our studies here. Calcium (Ca^+2^)-dependent (cytosolic) PLA2 (cPLA2), which has received the most attention [20]—and particularly the cPLA2 GIVA isoform—was examined. Small secreted PLA2 (sPLA2) enzymes have gained interest in neuroinflammatory models [21], among which sPLA2 GIIA received our focus. In contrast, Ca^+2^-independent PLA2 (iPLA2) isoforms may have brain homeostatic roles; we were principally interested in iPLA2 GVIA, because of its importance in the turnover of endogenous docosahexaenoic acid (DHA, 22:6n-3) [22]. Moreover, we were guided by our results from brain regions of adult rats given moderate rather than high ethanol doses in a “binge-pattern” [23], which showed reduced iPLA2 GVIA and significantly elevated expression levels of cPLA2 GIVA, sPLA2 GIIA, and AQP4, as well as poly (ADP-ribose) polymerase-1 (PARP-1). PARP-1 is a key nuclear DNA repair enzyme, but when hyperactivated, it can promote a form of regulated necrosis (termed parthanatos) by oxidative stress routes involving glial activation [24], [25].

The above neuroinflammation-related enzymes were examined in the Majchrowicz *in vivo* intoxication model, in parallel with a somewhat unique *in vitro* model, binge ethanol-treated adult-age rat organotypic hippocampal-entorhinal cortical (HEC) slices in culture. Organotypic brain slice cultures (typically hippocampal and only occasionally HEC) have often been used in neurodegeneration studies [26], [27], but invariably at adolescent or developing brain ages. It seemed reasonable that data with adult-age HEC slice preparations would more reliably correlate with results from intact adult brain. For example, the adult-age rat HEC slices incur neurodamage and AQP4 elevations when subjected to binging that closely approximates the *in vivo* model (four successive [overnight] exposures to 100 mM ethanol—a concentration not unusual in the blood of severe alcoholics [28], [29] that is frequently used in neurodegeneration studies—whereas developing HEC slice cultures require ∼6 exposure days) [30]. At least with entorhinal cortex, the above suggestion that adult brain neurons are more susceptible than developing neurons to binge ethanol-induced neurodegeneration corresponds well with binge ethanol results *in vivo* [31]. Since edema is linked at times to reactive oxygen/nitrogen species, we also assessed the extent of oxidative stress in the ethanol-binged slices using “oxidative footprints”—levels of 4-hydroxynonenal (4HNE)-adducted and/or 3-nitrotyrosinated (3NT-) proteins. In part, the latter assays were motivated by indications in other disease conditions involving brain oxidative stress, e.g., hepatic encephalopathy, that protein tyrosine nitration is associated with brain edema [32].

An important facet of this study is the investigation of the neuroprotective effects of DHA against ethanol-dependent brain damage [30], [33]. Endogenous rat brain levels of this essential n-3 unsaturated fatty acid are equal to those of n-6 ARA, but in contrast to the latter, DHA is often anti-oxidative and anti-inflammatory rather than pro-inflammatory [34]–[36]. Depletion of brain DHA, principally within membrane phosphatidylserine, could underlie aberrant neuroapoptosis in fetal alcoholism models [37], [38], and dietary DHA can counter the brain oxidative damage and neurobehavioral deficits of ethanol exposure in utero [39]–[41]. We find with the ethanol-binged adult-age HEC slice cultures that endogenous DHA is indeed reduced, and that supplemented DHA normalizes those levels, reduces oxidative stress, and exerts global suppression of the above neuroinflammatory proteins. One of these, PARP-1, may be a crucial regulatory enzyme in a pro-inflammatory phospholipid-dependent cascade.

## Materials and Methods

### Materials

#### Antibodies

AQP4 (sc-20812), cPLA2 IVA (sc-454), ser 505 p-cPLA2 IVA (sc-34391), and glyceraldehyde phosphate dehydrogenase (GAPDH, sc-166545) were from Santa Cruz Biotechnology, Santa Cruz CA; iPLA2 VIβ (07-169) was from Upstate Biotech, Lake Placid NY; sPLA2 IIA (181055100) was from BioVendor, Candler NC; PARP-1 (9542s) was from Cell Signaling, Danvers MA; Anti-3NT-protein (AB7048) was from Abcam Co., Cambridge MA; and anti-4HNE-adducted protein (AB5605) was from Millipore Corp., Temecula CA. Secondary antibodies were from Jackson ImmunoResearch, West Grove PA, and luminol reagent for immunoblot detection was from Pierce Chemicals, Rockford IL. DHA was from Cayman Chemicals, Ann Arbor MI, PJ34 was from Enzo Life Sciences, Farmingdale NY, and other chemicals/reagents were from Sigma Chemicals, St. Louis MO.

### Animal experiments

These studies were carried out in accordance with the recommendations in the Guide for the Care and Use of Laboratory Animals of the National Institutes of Health. The *in vivo* protocol was approved by the Institutional Animal Care and Use Committee of the University of Kentucky, and all efforts were made to minimize suffering during treatments and sacrifice. The *in vitro* protocol was approved by the Institutional Animal Care and Use Committee of Loyola University Medical Center, and all efforts were made to minimize suffering during anesthesia and sacrifice.

### Binge ethanol intoxication in vivo

Adult male SD rats (275–300 g) were subjected to the 4-day binge intoxication protocol modified from Majchrowicz that has a well-documented neurodegeneration profile [8], [42], [43]. The model mimics the high blood ethanol levels of binge pattern alcoholics [3]. Rats were provided nutritionally complete liquid diets containing either ethanol (25%w/v) or isocaloric dextrose in vanilla Ensure Plus (Abbott Laboratories, Columbus OH) every 8 hr via intragastric gavage (range: 8.3–9.7 g/kg/d). Rats in the ethanol group received an initial loading dose of 5 g/kg, with subsequent doses adjusted based on a behavioral intoxication scale detailed in earlier reports [44], [45]. Control rats were given volumes of diet equal to the average volumes given the ethanol-treated rats. Water was freely available but not chow. Serum ethanol levels, which averaged ∼375 mg/dl (∼82 mM), were ascertained in blood samples taken 90 min after the 6^th^ ethanol dose using an AM1 Alcohol Analyzer (Analox, London UK) calibrated against a 300 mg/dl external standard. Rats were sacrificed by decapitation on the morning of the 5^th^ day, 8–10 hr after the last ethanol or control gavage treatment. Intact brains were quickly removed, divided in half and quick-frozen in liquid nitrogen. At appropriate times, brains were thawed on ice and respective brain regions were taken for protein extraction as described under Immunoblotting.

### Adult-age HEC slice cultures

Male Sprague-Dawley rats (Harlan, Indianapolis IN), 40±2 days old, were anesthetized with isoflurane and thoroughly perfused *via* the intracardiac route with ∼150 ml of ice-cold lactate-Ringer's solution. When translucent, rat brains were placed in ice-cold Gey's buffer solution; the hippocampal-entorhinal cortical complex was removed and slices (250–300 µ) were prepared using a McIlwain tissue chopper. Slices were carefully placed on Millipore 0.4 µ membrane inserts (2–3 slices/insert), which were placed in 6-well plates, with each well containing 1.2 ml of MEM media with 25% heat-inactivated horse serum (HS). Slices were incubated at 32°C and 5% CO2 for the first 48 hr, and media was then changed to 20% heat-inactivated HS, and slices were transferred to a 37°C incubator (5% CO2). Cultures were then maintained 17–19 days, with the media changed every 2–3 days. Slices were monitored visually and those appearing unusually dark or broken were discarded.

### Binge ethanol and DHA treatment of HEC slice cultures

At a brain age of 60±3 days, slices in experimental groups were treated overnight for ∼16 h with 100 mM ethanol in MEM/20% HS for 4 successive nights, media without ethanol for ∼8 h during daylight hours for 3 d and then ∼4 h on the 4^th^ day, to permit slice assays. Control slices were subjected to similar changes throughout the 4 d period with HS-containing MEM media without ethanol. Half of experimental and control slices were treated with DHA (25–50 µM) or PJ34 (10 µM) beginning 4 h prior to the start of ethanol treatment and throughout both overnight ethanol/media and daytime media exposures. The DHA concentrations used, reported to sustain neuronal viability in primary brain cultures [46], border physiological values for n-3 fatty acids [47]. Slices then were treated as described below with propidium iodide (PI), a fluorescent dye selectively accumulated by dying and dead neurons in organotypic brain slices [48], [49], or pooled for immunoblot analyses.

### PI staining for assessment of neurodegeneration in HEC slices

Media were removed from the wells and slices were incubated for 20–30 min with 1.2 ml fresh serum-free media containing 10 µM PI. After removal of PI-containing media, slices were washed twice with serum-free media, followed by addition of complete media with 20% HS for 1 hr before observing fluorescence results of PI labeling.

### Immunoblotting with rat brain tissues

For HNE- and 3NT-proteins in brain regions of binge ethanol-treated and control rats and in pooled HEC slices, isolated brain regions or slices (rinsed with phosphate-buffered saline (PBS) and pooled—6-9 slices) were lysed in an isotonic buffer (50 mM Tris, 1 mM EDTA, protease and phosphatase inhibitor cocktail, pH 7.4), and then centrifuged for 10 min @ 13,000 rpm. For the neuroinflammation-associated proteins (cPLA2, p-cPLA2, sPLA2, iPLA2, AQP4, PARP-1), isolated brain regions or slices as above were sonicated 15–20 sec in a different lysis buffer (0.1% SDS, 1% sodium deoxycholate, 1% triton X-100, 150 mM NaCl, and 25 mM Tris HCl, pH 7.6) containing protease and phosphatase inhibitor cocktail. After centrifugation as above, protein concentrations were acquired with a bicinchoninic acid (BCA)–based method (Pierce Biotech, Rockford, IL); Aliquots (10 µg protein) of supernatants were then separated by 12% SDS-PAGE, transferred to PVDF-immobilon membranes, and subjected to immunoblot analysis by using appropriate protein antibodies. The intensities of immunoblots were normalized to GAPDH immunoblots, obtained on the same gels following stripping and reprobing, after scanning images with LABwork 4.5 image acquisition and analysis software (UltraViolet Products, Upland CA).

### Unsaturated fatty acid assay

Lipids were extracted in the presence of internal standard (IS), tricosanoic acid (23:0), following established procedures in the Kim laboratory [50]. Aliquots were transmethylated by reaction with BF_3_-methanol (14%, w/v) at 100°C for 2 h under nitrogen, and the methyl esters were then extracted with hexane and analyzed by gas chromatography-flame ionization detection. Individual fatty acids were identified by comparing retention times with known fatty acid standards (GLC-411 from Nu-Chek Prep., Elysian MN, USA). After comparing peak areas to IS in the conventional way, the contents of individual fatty acids were expressed as percent of total identified fatty acids [50].

### Statistical analyses

Unless indicated otherwise, results were reproduced with 6–15 brain slices per group in each experiment. Quantitation of PI fluorescence with 1.99 NIH Image J software was as previously described in detail [33]. For the protein extracts of both *in vivo* brain regions and HEC slices, immunoblot levels were determined relative to levels of GAPDH as housekeeping protein, and expressed as percent of control (mean ± sem). Results were analyzed for statistically significant differences (p<0.05 or p<0.01) by Tukey's t-test and one-way analysis of variance (ANOVA) with completely randomized design.

## Results

### In vivo study

**Figure 1:**
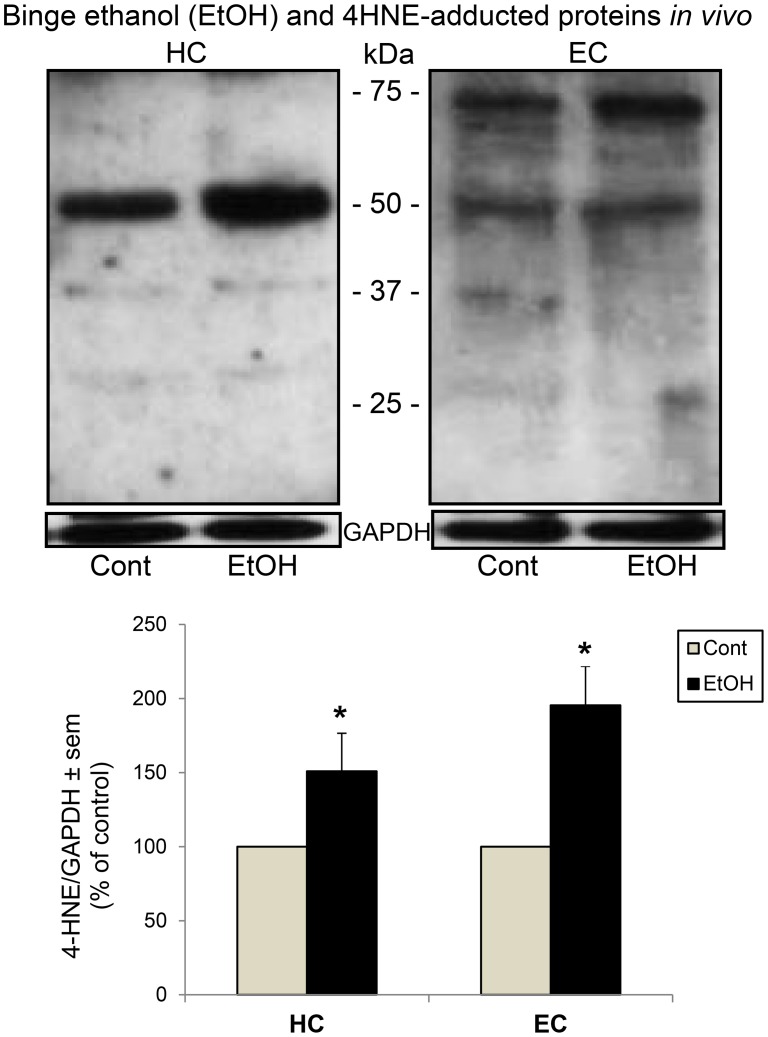
Effects of binge ethanol exposure on oxidative stress (4HNE-) protein footprint levels *in vivo*. Significant increases over respective controls (Cont) in levels of oxidative stress footprint, 4-hydroxynonenal (4HNE)-adducted proteins, in hippocampus (HC) and entorhinal cortex (EC) following neurotoxic binge ethanol treatment of adult male rats for 4 days (Majchrowicz model). Top: representative immunoblots of 4HNE-adducted proteins in HC and EC. Bottom: quantitation of immunoblots of 4HNE-adducted proteins in HC and EC. *p<0.01 vs. control (Cont); n = 4–7 rats per group.

Figure 1 confirms the presence of substantial oxidative stress, i.e., 4HNE-adducted proteins that arise from peroxidation of endogenous/released AA and adduction of the peroxidative aldehyde product, 4HNE, with protein sidechains [51], in two susceptible brain regions *in vivo* (Majchrowicz rat model) after 4 days of repetitive ethanol gavages and morning sacrifice on day 5. It is very likely that the large immunoblots in the range of 50–55 KDa for both regions and also ∼70+ KDa in EC constitute not just single proteins but an appreciable number of 4HNE-adducted proteins, based on 2-dimensional gel studies with such adducts [52]. When all discernible 4HNE-immunoblots are combined in the quantitation, significant elevations in levels of 4HNE-adducted oxidative footprints in HC and EC are observed, consistent the reported effectiveness of antioxidants in reducing neurodegeneration in this in vivo model [53].

**Figure 2:**
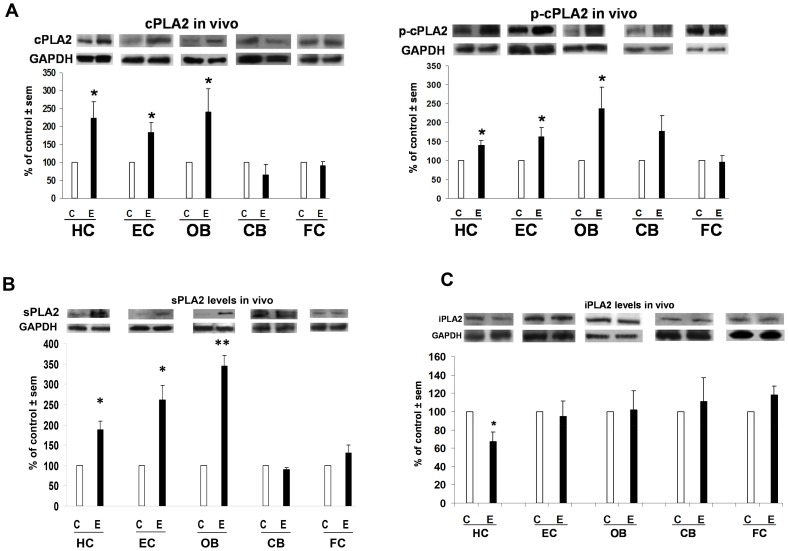
Selective effects of binge ethanol exposure on brain PLA2 levels *in vivo*. Significant alterations compared to respective controls (C) in protein expression (immunoblots) of PLA2 enzymes in brain regions selectively incurring neurodamage due to binge ethanol (E) treatment in adult male rats for 4 days (Majchrowicz model). Fig. 2A: Significantly increased levels of cPLA2 (top) and p-cPLA2 (bottom) in the hippocampus (HC), entorhinal cortex (EC) and olfactory bulb (OB), but not in cerebellum (CB) and frontal cortex (FC). Fig. 2B: Significantly increased sPLA2 levels in the HC, EC and OB, but not in CB and FC. Fig. 2C. Significantly decreased levels of iPLA2 in the HC. *p<0.05 vs. C. **p<0.01 vs. C. n = 4–7 rats/group.

**Figure 3:**
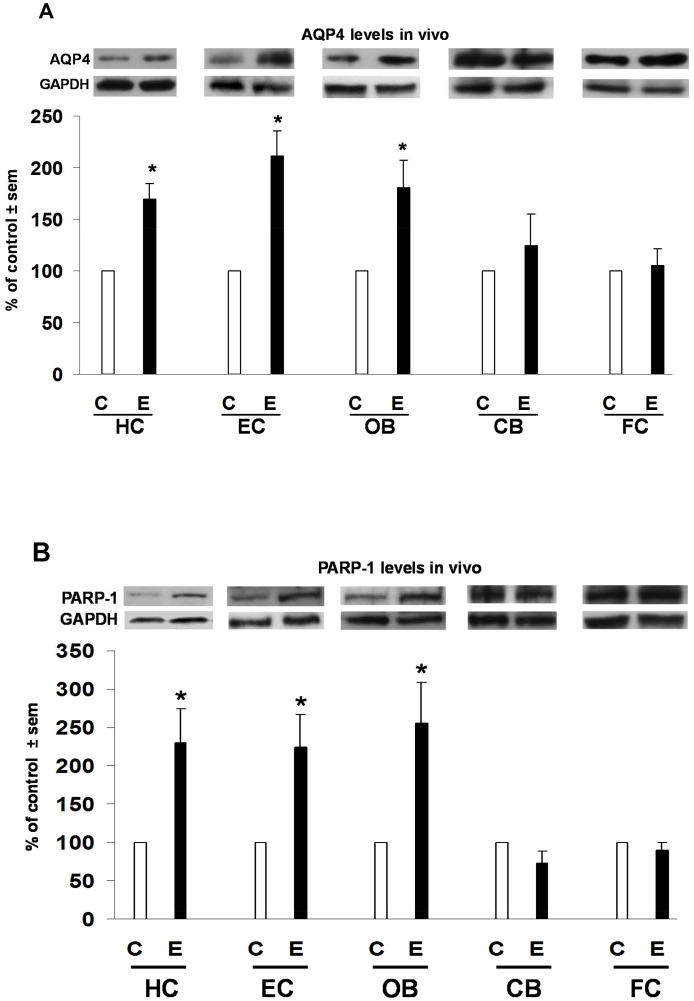
Selective effects of binge ethanol exposure on brain AQP4 and PARP-1 levels *in vivo*. Significant elevations over respective controls (C) in levels of AQP4 and PARP-1 in those brain regions selectively incurring neurodamage due to binge ethanol (E) treatment in adult male rats for 4 days (Majchrowicz model). Fig. 3A: Significantly increased AQP4 levels in the HC, EC and OB, but not in CB and FC. Fig. 3B: Significantly increased PARP-1 levels in the HC, EC and OB, but not in CB and FC. *p<0.05 vs. C. n = 4–7 rats/group.

Figures 2 and 3 are representative immunoblots of the neuroinflammation-associated proteins—cPLA2, p-cPLA2, sPLA2, iPLA2, AQP4 and PARP-1—and their *in vivo* levels, normalized to GAPDH, in five brain regions of adult male rats after neurotoxic binge ethanol or control treatments. Figure 2A shows that levels of cPLA2 and its phosphorylated/activated form, p-cPLA2, were significantly increased 40–140% above controls in the three regions that sustain appreciable neurodegeneration in the model—HC, EC and OB [8]. In contrast, cPLA2 and p-cPLA2 levels did not differ from control values in the CB and FC, two regions typically having little or no neurodamage. In Fig. 2B, the regional changes in sPLA2 mirrored cPLA2—significant elevations ranging from 75% to 240% above control levels in the three vulnerable regions of HC, EC and OB, but no significant changes in CB or FC. Figure 2C shows that iPLA2 was affected differently by binge ethanol treatment, being significantly reduced 35% below control values in the HC. It was not appreciably changed from control values in the other brain regions.

Figure 3 shows that *in vivo* responses to neurotoxic ethanol binging for AQP4 and PARP-1 mirrored those of cPLA2, p-cPLA2 and sPLA2. As with the cPLA2 and sPLA2 families, ethanol binges significantly increased AQP4 levels 65–110% above controls in the HC, EC and OB, whereas levels of the water channel did not differ from controls in the CB and FC. As shown in Figure 3B, PARP-1 also corresponded with the AQP4, cPLA2, p-cPLA2 and sPLA2 results. Quantitation of PARP-1 immunoblots revealed significant elevations of 125–150% above controls in the neurovulnerable regions of HC, EC and OB, but there were no changes in its levels in CB and FC.

### In vitro studies

**Figure 4:**
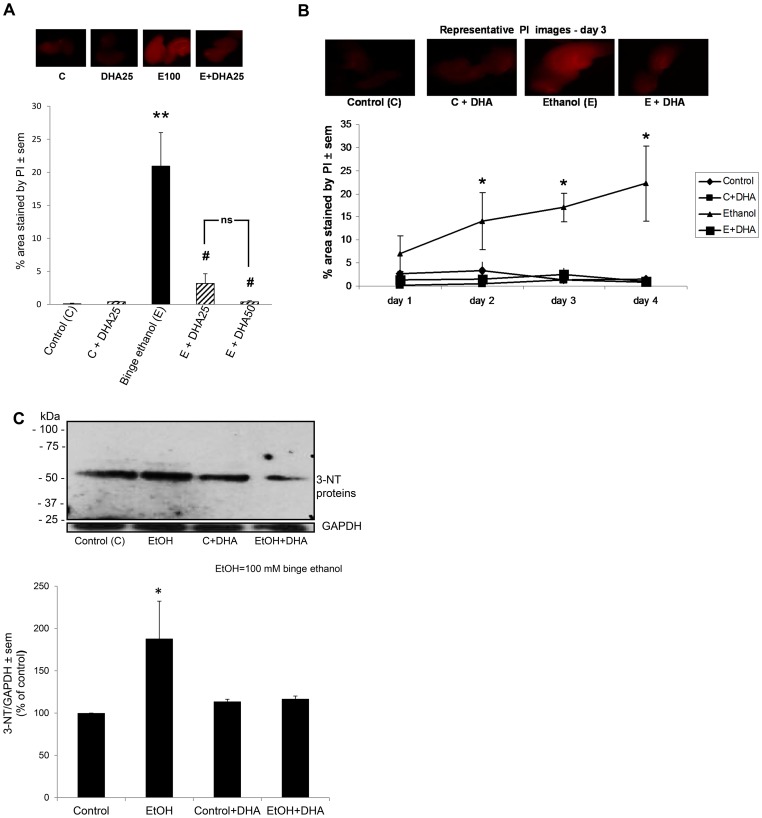
Effects of binge ethanol treatment and DHA supplementation on neurodegeneration and oxidative (3NT-) protein footprints in adult-age HEC slice cultures. Binge ethanol treatment of adult-age rat HEC slice cultures for 4 days as described in Methods significantly increases degenerating neurons (PI staining) and the levels of oxidative stress footprints (3NT-proteins), and supplementation of cultures with DHA prevents the increases. Fig. 4A: (top) Representative PI-stained images showing increased neurodegeneration after 3 days of binge ethanol treatment (100 mM), with DHA supplementation (25 µM) suppressing PI staining (neurodegeneration). (Bottom) Quantitation of PI labeling shows significant neurodegeneration due to 4 days of binge ethanol exposure (E), and neuroprotection against E by DHA at 25 and 50 µM. **p<0.01 vs. C. #p<0.05 vs. E. Fig. 4B. Quantitation of PI labeling reveals significantly increased neurodegeneration over control due to binge E as early as 2 days of binge ethanol treatment, with prevention of the neurodegeneration throughout the 4 days of treatment by DHA supplementation. *p<0.05 vs. control (C) or E+DHA. Fig. 4C (Top) Representative immunoblots of 3NT-proteins in HEC slice cultures following binge ethanol exposure (100 mM) for 4 days. (Bottom) Quantitation of immunoblots showing that binge ethanol exposure causes increased 3NT-proteins in the HEC slice cultures, and DHA supplementation (25 µM) prevents the increases. *p<0.05 vs. Control.

**Figure 5:**
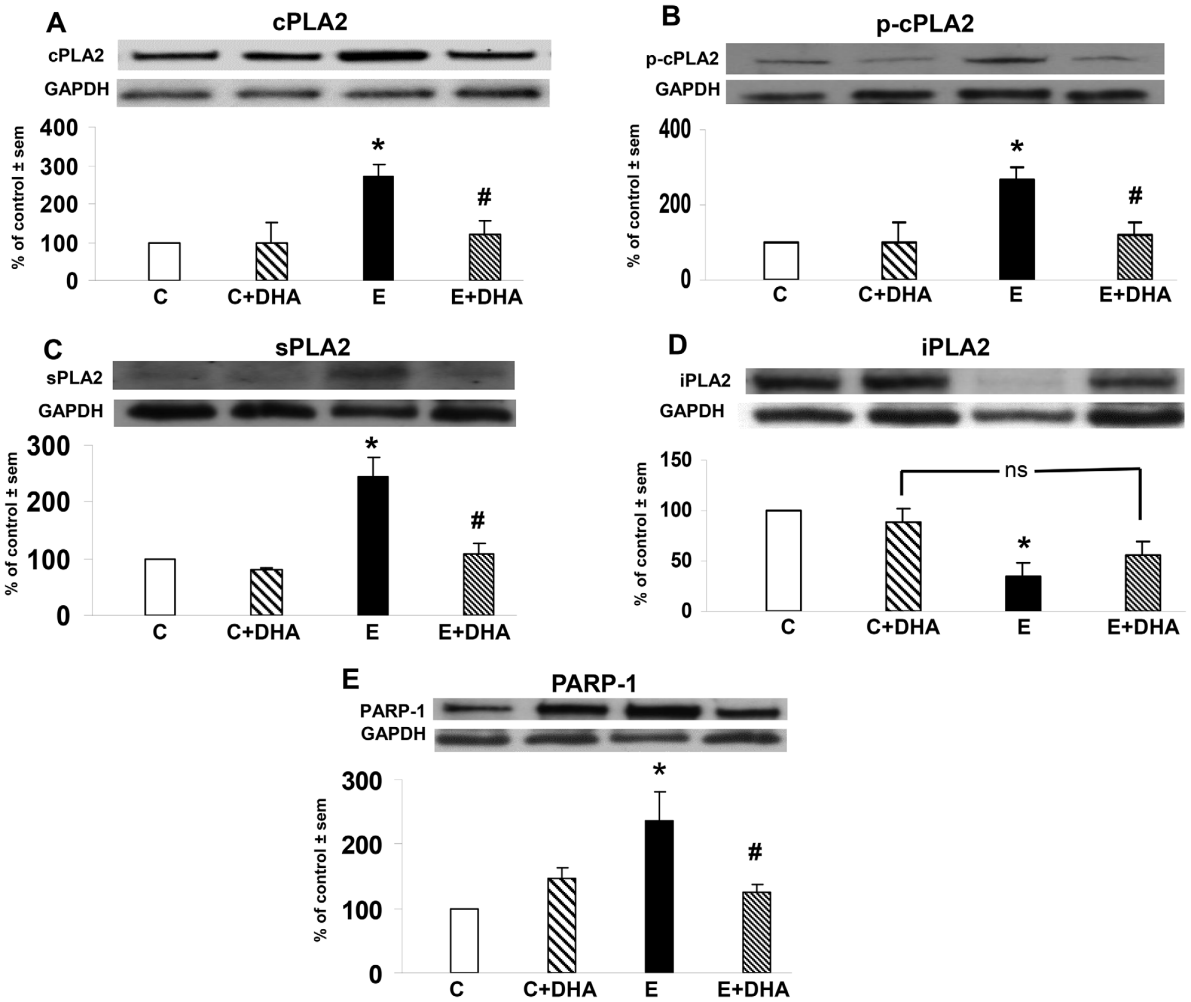
Effects of binge ethanol treatment and DHA supplementation on PLA2 and PARP-1 levels in adult-age HEC slice cultures. Significant neuroinflammatory enzyme alterations compared to control (C) caused by 4 days of binge ethanol (E) treatment (100 mM) of adult-age rat HEC slice cultures, and prevention of the alterations by supplementation of cultures with DHA (25 µM). Fig. 5A: Increased levels of cPLA2 GIVA over control levels due to binge E and inhibition of the increase by DHA supplementation (E+ DHA). *p<0.05 vs. C; #p<0.05 vs. E. Fig. 5B: Increased levels of p-cPLA2 GIVA over control levels due to binge E and inhibition of the increase by DHA supplementation (E+DHA). *p<0.05 vs. C; #p<0.05 vs. E. Fig. 5C: Increased levels of sPLA2 GIIA over control levels due to binge E and inhibition of the increase by DHA supplementation (E+DHA). *p<0.05 vs. C; #p<0.05 vs. E. Fig. 5D: Reduced levels of iPLA2 GVIA with respect to control levels due to binge E and partial blockade of the reduction by DHA supplementation (E+DHA). *p<0.05 vs. C. Fig. 5E: Increased levels of PARP-1 over control levels due to binge E and inhibition of the increase by DHA supplementation (E+DHA). *p<0.05 vs. C; #p<0.05 vs. E.

**Figure 6:**
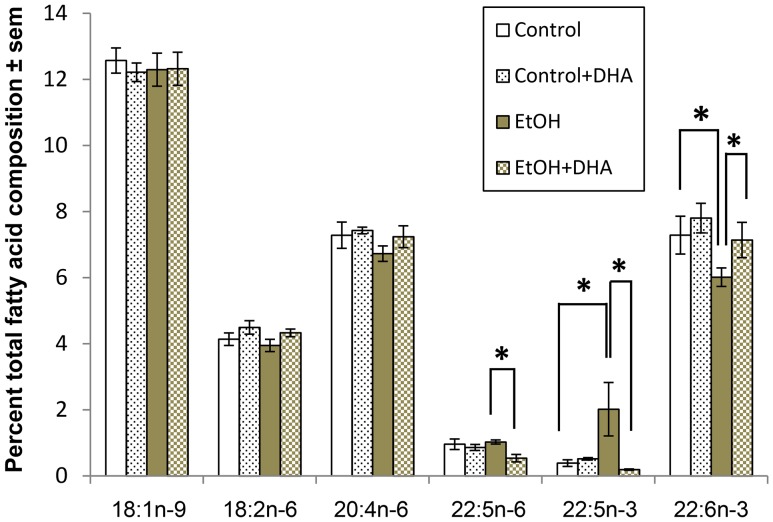
Effect of binge ethanol treatment and DHA supplementation on contents of DHA and selected unsaturated fatty acids in adult-age HEC slice cultures. Binge ethanol treatment (EtOH, 100 mM) of adult-age rat HEC slice cultures for 4 days as described in Methods significantly decreases the slice content of endogenous DHA (22:6n−3); supplementation of ethanol-binged cultures throughout with 25 µM DHA normalizes the DHA content. Binge ethanol treatment also increased the endogenous content of 22:5n−3, and DHA supplementation reduced the levels of this potential DHA precursor, as well as reduced the endogenous levels of ARA (22:4n−6). All other unsaturated fatty acids (not shown) were not significantly changed by binge ethanol treatment and DHA supplementation. *p<0.05 (n = 3/group, Tukey's t-test).

Figure 4, Figure 5 and Figure 6 demonstrate the effects in the rat adult-age HEC slice cultures of binge ethanol exposure on key neurotoxic outcomes, the above neuroinflammation-associated enzymes, and specific unsaturated fatty acid levels, and moreover, the effects of DHA supplementation on these measures. Figure 4A (top) shows representative images of HEC slices from control, DHA (25 µM), ethanol and ethanol+DHA groups at the 3 day treatment timepoint showing intensified PI labeling of degenerating neurons due to binge ethanol, and the relative absence of PI-stained neurons in the ethanol+DHA co-treated slice. The bottom graph shows that DHA concentrations of 25 and 50 µM potently inhibit binge ethanol-induced PI labeling (degenerating neurons), and to approximately the same extent. In Figure 4B, the quantitation of the time course of PI-stained neurons over 4 days of binge ethanol treatment demonstrates that degenerating neuron levels rose significantly above control levels on day 2, and continued to moderately increase for the two remaining days. The co-presence of DHA effectively prevented the increased neurodegeneration in HEC slices on each of those three ethanol exposure days. Although not used in these DHA experiments, adrenic acid, an analogous 22-carbon n-6 fatty acid and a negative control which was previously added to HEC slice cultures in place of DHA, had no effect on binge ethanol-dependent neurodegeneration [33].

In Figure 4C the results show that binge ethanol treatment of the HEC slice cultures for 4 days significantly augmented oxidative stress as indicated by elevations above basal levels in 3NT-proteins derived from reactions of peroxynitrite (product of the free radicals, superoxide and nitric oxide) with proteins. As with the Figure 1 oxidative protein footprints, the large immunoblots contain a number of proteins with nitrotyrosine sidechains that react to antibody, and quantitation encompassed all observable immunoblots. Consistent with its effects on neurodegeneration, co-exposure of ethanol-binged slices with DHA (25 µM) largely abolished elevations in this oxidative footprint. Parenthetically, we found that HNE-adducted proteins as in the *in vivo* brain samples could not be reliably determined in the slices because of interference from aldehydic adducts derived from supplemented DHA.

Figure 5 displays the immunoblot levels of five of the six neuroinflammation-related proteins (i.e., in Figures 2 and 3) in adult-age HEC slice cultures that were binge-exposed for 4 days to ethanol (100 mM) or control media, with and without DHA supplementation. AQP4 augmentation by binge ethanol in these adult-age slice cultures and suppression by added DHA were previously communicated [30]. Here it is evident that neurotoxic binge ethanol treatment caused significant elevations (160–240% above controls) in levels of cPLA2 (Figure 5A) and p-cPLA2 (Figure 5B), the latter confirming cPLA2 activation. Whereas added DHA alone had no effects relative to controls, its supplementation throughout ethanol treatment prevented increases in both proteins. Similarly, in Figure 5C, HEC slice sPLA2 levels were potentiated ∼150% above controls by the binge ethanol exposure; as with cPLA2, added DHA only did not change sPLA2, but supplementation abolished ethanol-induced sPLA2 elevations. With iPLA2 (Figure 5D), consistent with *in vivo* HC results in Fig. 2C, its levels in the HEC slices were significantly reduced ∼65% by binge ethanol. DHA alone had no effect on iPLA2 levels, but its supplementation with binge ethanol countered the reduction in iPLA2, thus normalizing its levels. In Figure 5E, also coinciding with *in vivo* findings, PARP-1 levels were significantly enhanced ∼130% over control values by the binge ethanol exposure. DHA alone did not alter PARP-1 levels, but consistent with DHA's PLA2 and AQP4 suppression and its prevention of neurodegeneration (Figure 4), co-treatment with the n-3 fatty acid nullified binge ethanol's potentiation of PARP-1 levels.

Figure 6 shows quantitative results of assays of selected endogenous long-chain unsaturated fatty acids in lipid extracts of the binge ethanol-exposed adult rat HEC slices, with and without DHA supplementation. Expressed as percent of total fatty acids, endogenous brain content of DHA (22:6n−3) was significantly depleted by ethanol exposure, and supplementation with DHA (25 µM) prevented the depletion—results agreeing with earlier *in vivo* findings in the Kim laboratory. Concomitantly, binge ethanol treatment significantly increased a likely DHA precursor, 22:5n−3, with DHA supplementation promoting a normalization of this fatty acid's contents.

**Figure 7:**
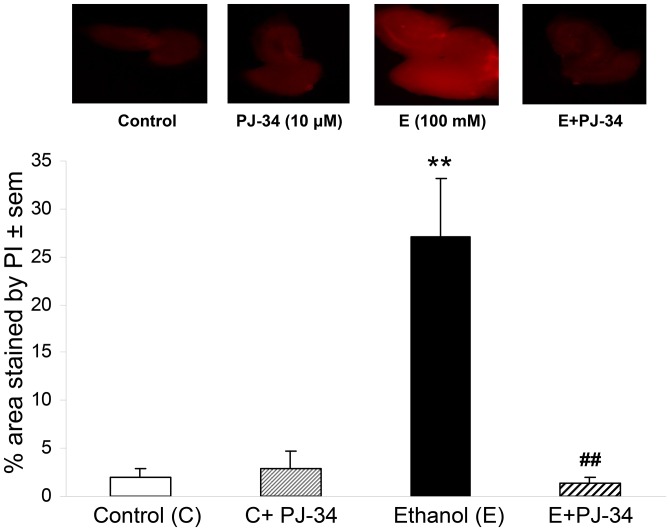
Effect of PARP-1 inhibitor PJ-34 on ethanol-induced neurodegeneration in adult-age HEC slice cultures. Neurodegeneration (PI staining) due to 4 days of binge ethanol treatment (100 mM) of rat adult-age organotypic HEC slice cultures is suppressed by co-treatment with PARP-1 inhibitor, PJ-34 (10 µM). (Top) Images of representative PI-stained slices from C, PJ-34, E and E+PJ-34 slice cultures indicate increased neurodegeneration (E) that is reduced in E+PJ-34. (Bottom) Quantitation of PI staining demonstrates increased neurodegeneration due to binge ethanol (E) that is significantly suppressed by PJ-34 (E + PJ-34). **p<0.01 vs. C. ##p<0.01 vs. E.

Interested in the possibility that PARP-1, known to be activated by oxidative stress and DNA strand breaks, could have direct upstream actions on ethanol-induced neurodegeneration in the adult-age organotypic HEC slices, we used the relatively specific PARP-1 inhibitor PJ-34 [54] at 10 µM for the initial assessment. In Figure 7 (upper) are representative images of HEC slices showing increased PI staining (neurodegeneration) after 4 days of binge exposure to ethanol (100 mM) without PJ-34, and apparent suppression of fluorescence by PJ-34 co-administration. Quantitation in Figure 7 (lower) of fluorescence verified significant neurotoxicity in ethanol-binged slices, and while PI staining with PARP-1 inhibitor PJ-34 alone was not different from controls, its co-presence exerted significant neuroprotection against the ethanol-induced neurotoxicity.

## Discussion

We demonstrate with age-complementary *in vivo* and *in vitro* adult rat brain models that major neuroinflammation-associated pro-oxidative pathways encompassing AQP4, key PLA2 families, and PARP-1 are significantly altered in binge ethanol-dependent neurodegeneration. Specifically, neurotoxic ethanol binging promotes significant elevations in cPLA2, p-cPLA2, and sPLA2 and decrements in iPLA2 in both models. The *in vivo* results with the well-documented “Majchrowicz” binge model [6] were distinguished by the fact that, along with AQP4 and PARP-1, the above PLA2 elevations occurred in three brain regions that typically sustain neurodegeneration, but not in two regions having few if any degenerating neurons. In adult-age rat organotypic HEC slice cultures the ethanol-induced changes in these neuroinflammation-linked proteins agreed well with the *in vivo* findings, and furthermore, were in accord with recent binge ethanol results *in vivo* [45] in that the neurodegeneration becomes manifested within a few days of exposure. Whether the above proteins constitute a relatively unified cohesive pathway or are components of interacting, even redundant, signaling routes for induced oxidative neurodamage is yet to be established; however, the results (Figure 7) with a PARP-1 inhibitor provide preliminary evidence implicating a PARP-1-contingent neurotoxic process. The results further indicate that supplementation with an essential n-3 fatty acid, DHA—previously reported to halt ethanol-engendered neurodegeneration in binge-treated brain slices—additionally suppresses alterations in the neuroinflammatory proteins and oxidative stress footprints.

Among the proteins examined, AQP4 is of particular interest, as research principally with ischemic or traumatic brain damage models, but also with water intoxication and certain brain infections, demonstrates that increased activity or levels of the astroglial water channel regulates cytotoxic (cellular) brain edema [17], [55]. Astroglial overexpression of AQP4 in mice accelerates ischemia-induced brain edema [56], and AQP4 knockdown in traumatized astroglial cultures reduces cell swelling [57]. AQP4 may have a prominent neuroinflammatory role in brain insults, since experiments with AQP4 knockout mice confirm the protein's requirement for full induction of brain proinflammatory cytokines (notably tumor necrosis factor alpha) during endotoxin treatment or experimental allergic encephalomyelitis [58]. Thus AQP4 acts as a brain neuroinflammatory channel during particular stresses or insults.

After finding moderate brain edema in a once-daily adaptation of the Majchrowicz ethanol gavage model, we determined that edema's deterrence with furosemide in that model and in ethanol-binged HEC slice cultures was neuroprotective [11]—as it was with the diuretic, acetazolamide [12], which fortuitously is an AQP4 inhibitor. Astroglial cells in culture swell due to ethanol exposure and withdrawal, but previous evidence of blockade by diuretics has been conflicting [59], [60]. However, acceleration of trauma-related cerebral edema by ethanol has been linked to increased AQP4 upregulation and activity, with acetazolamide, presumably through inhibition of AQP4, exerting protection [61]. Herein, binge ethanol-induced AQP4 elevations in brain regions that sustain neurodegeneration, but not in regions known to lack neurodamage, indicate that the water channel could be an important factor in the regioselective neurodegeneration. Furthermore, increased AQP4 in the binge-treated adult-age HEC slice cultures, as well as being in accord with *in vivo* results, is in harmony with our findings in developing HEC slice cultures [12]. How AQP4 levels are altered by ethanol binges requires study, but several stimuli known to potentiate brain or astroglial AQP4 levels/activity are reported by others to be increased by ethanol in various exposure protocols. For example, ROS [62], lactic acid [[63], proinflammatory cytokines [64], and glutamate [65] have been shown to increase the water channel. Depending on the model, ethanol and/or ethanol withdrawal can promote or potentiate brain levels of all of these factors [9], [66], [67].

PLA2 isoforms, and chiefly those of Ca^+2^-dependent cPLA2 and sPLA2 families, are rate-controlling for normal or excessive release of ARA. This essential n-6 fatty acid is precursor for neuroinflammatory eicosanoids as well as ROS generation *via* subsequent action of cyclooxygenases and/or autooxidation. Interestingly, a feed-forward mechanism could also operate, since ARA-derived eicosanoids (leukotrienes) can increase AQP4 and promote edema [68]. Of the two PLA2 families, cPLA2 (∼85 kDa) and its hyperactivation have been most frequently linked to AA-derived neuroinflammation and neurodegeneration [20], [69]. Furthermore, PLA2 isoforms are prone to be increased and/or activated by brain cell swelling and deformation [18], and cPLA2 can act as a mechanosensitive transducer to cellular osmotic stress and membrane stretching [70], [71]. Also, phosphorylation by mitogen-activated protein kinase (MAPK) family members, a catalytic step producing activated p-cPLA2, is similarly modulated by osmotic or stretch-induced stress [72], [73], which is entirely compatible with the mechanisms in these binge ethanol models.

The smaller sPLA2 family members (13–20 KDa), either independently or complicit with cPLA2, could be involved in brain neuroinflammatory mechanisms [74]. Notably, inhibition of sPLA2 has been found to reduce neuroinflammation and ischemic injury in adult rats [75]. Brain sPLA2 IIA elevations have been documented in rat stroke models [76], during Alzheimer's disease [77], and now in the acquired brain damage caused by binge ethanol in organotypic brain slice cultures. However, other sPLA2 family members such as sPLA2 V and most recently sPLA2 III, expressed intraneuronally in synaptic regions [78], also could be important in ethanol's mechanism and should be investigated. It has been known for some time that chronic ethanol in vivo increases brain PLA2 activity in general [79], so at this juncture it is plausible to consider that the elevated cPLA2 and sPLA2 activities are components of downstream neuroinflammatory activation and ROS increases during binge ethanol exposure.

Furthermore, the observed reduction in iPLA2 GVI levels due to ethanol is in harmony with neuroinflammatory outcomes that indicate a reciprocal relationship between iPLA2 and cPLA2/sPLA2 [80], [81]. This could relate to iPLA2 possibly serving as a mitochondrial housekeeping and protective enzyme [22]. Studies have associated iPLA2 with suppression of lipid peroxidation [82], and such an anti-oxidant function could be explained by the enzyme's activity in sustaining endogenous DHA turnover in brain membrane phospholipids [83]. Reduced DHA turnover related to iPLA2 depletion in binge ethanol-exposed brain could portend diminished endogenous neuroprotection associated with this n-3 fatty acid (discussed in more detail below).

Binge ethanol's significant potentiation of multi-faceted PARP-1 selectively *in vivo* as well as in HEC slices, in concert with a PARP-1 inhibitor's suppression of neurodegeneration (and also our previous report with a relatively moderate binge rat model for robust PARP-1 augmentation [23]), support a role in ethanol neurotoxicity for this previously underappreciated player. Nuclear PARP-1 responds to oxidative stress and DNA strand breaks to synthesize polyADP-ribose (PAR) chains that then recruit DNA repair protein assemblies. However, PARP-1 hyperactivation can deplete essential cellular NAD+ levels and ATP, as well as trigger PAR-directed mitochondrial non-apoptotic death, which is termed parthanatos [24]. Parthanatos is a probable neuronal death pathway in a transgenic Parkinsonian mouse model [84] and in endotoxin-induced neuroinflammation [85]. To our knowledge, PARP-1-dependent neuronal demise has not been linked convincingly to ethanol, nor has the enzyme been experimentally associated with brain AQP4- or PLA2-dependent neuroinflammatory pathways. However, a functional link between PARP-1 activity and binge ethanol neuropathology as indicated by PJ-34 inhibitor results (Figure 6) is consistent with elevated brain oxidative stress in chronic ethanol-treated rodents [9], oxidative DNA damage in brain of alcoholics [86], and—in regard to parthanatos—the absence of neuronal apoptosis (i.e., predominance of necrosis) in the Majchrowicz binge ethanol model [10]. Future experiments with the binge models should employ RNA interference and other inhibitors together with PAR measurements in order to more firmly establish PARP-1's role in binge ethanol-induced neurodegenerative signaling.

The prominent actions of supplemented DHA in the adult HEC slices may provide insights into neuroinflammatory protein mechanisms in the binge ethanol models. Growing evidence indicates that DHA administration during other neurodamaging conditions or diseases can promote prosurvival and/or neuroprotection [87], [88]. The data herein show that DHA supplementation of binge ethanol-treated HEC slice cultures inhibits elevations in the selected PLA2 families, PARP-1, and as reported, AQP4 water channels [30], concomitant with inhibiting neurodegeneration. To be noted is that DHA's suppression of these neuroinflammatory pathways *in vitro* is highly consistent with protective findings previously reported with the Majchrowicz binge intoxication model—(1), with furosemide, a diuretic having anti-oxidative and potentially anti-inflammatory effects [89]; (2) with cannabidiol, an anti-inflammatory compound that also exerts neuroprotection [89]; and (3) with metabotropic glutamate receptor stimulation, which selectively elevates transforming growth factor-β (suppressed by the binging) and inhibits entorhinal cortex neurodegeneration [90].

We consider that two overall neuroprotective mechanisms for DHA in the experimental literature could be functioning. The first advances that enriched esterification in the 2-position of phospholipids with DHA, and in particular, inner neuronal membrane phosphatidylserine, sustains neuronal survival against insults. In part such phosphatidylserine enrichment with DHA favors translocation and activation of PKB/Akt, a key pro-survival kinase that antagonizes oxidative signaling [91], [92]. Indeed, as Figure 6 demonstrates, gas chromatography assays of the HEC slice cultures reveal relatively selective reductions in endogenous DHA levels from binge ethanol treatment, with restoration by supplemented DHA.

A second mechanism relates to unesterified or “free” DHA and its metabolic products. Considerable attention has focused on DHA's dihydroxylation initiated by 15-lipoxygenase-1 to generate 10R,17S-dihydroxydocosa-4Z,7Z,11E,13E,15Z,19Z-hexaenoic acid or neuroprotectin-1 (NPD1); other oxidized isomers, inclusively termed resolvins, also may be produced *via* this or a related enzyme [93], [94]. NPD1, formed primarily in brain astroglia from DHA, is a highly potent cytoprotectant and antioxidant against retinal and brain ischemic insults, and can suppress brain neuroinflammation [95]. Another potentially neuroprotective product of nonesterified DHA that has received recent attention is amidated docosahexaenoylethanolamide (synaptamide) [96], which is structurally analogous to the arachidonic acid-derived cannabinoid, arachidonoylethanolamide (anandamide). Synaptamide has been shown to promote neurite growth and preserve synaptic function [97], and it is tenable that it, like the hydroxylated DHA derivatives, NPD1 and other resolvins, might wield antioxidant-related neurosurvival effects.

Integrating our findings within a mechanistic scheme, the speculation is that the first ethanol binges trigger limited but still effectual increases in ROS primarily during withdrawals (or its nadirs in vivo), somewhat akin to ischemia-reperfusion. Possible ROS sources include NADPH oxidase stimulation, ethanol metabolism (cytochrome P450, catalase, peroxidases), and/or mitochondrial membrane damage/leakage. Such early ROS generation could be sufficient to boost AQP4 expression via unspecified oxidative transcription factors, while also initiating nuclear DNA fragmentation and thus enhancing upregulation of PARP-1. Repetitive binges would greatly augment these events, leading to astroglial swelling-related activation/expression of cPLA2 and sPLA2, which evoke excessive AA mobilization, loss of endogenous DHA turnover due to decreased iPLA2, and greater increases in neuron-damaging ROS levels, concomitant with PAR-mediated glial activation [54] and associated parthanatos.

Supplemented DHA, exerting anti-oxidant actions through routes outlined above, could overcome immediate ROS generation triggered by the first several ethanol binges and basically inhibit the cascade of downstream ROS-augmenting cascades that depend upon PARP-1, AQP4, and PLA2. Relevant to this scheme is that DHA co-treatment in a DNA alkylating agent-treated hippocampal cell line blocks PARP-1 activity (PAR formation) while preventing oxidative stress and neuronal death [98]. In view of PET neuroimaging results indicating DHA metabolic deficits in brains of chronic alcoholics [99], our DHA findings *in vitro* reinforce an implication from that report that n-3 fatty acid supplementation during withdrawal therapy could be beneficial for cognitive support and even neuroprotection. Indeed, enrichment of neuronal cell phospholipids and particularly phosphatidylserine with supplemental DHA has been known for some time to effect neuroprotection against a range of insults [100].
